# Development and validation of a bovine macrophage specific cDNA microarray

**DOI:** 10.1186/1471-2164-7-224

**Published:** 2006-09-01

**Authors:** Kirsty Jensen, Richard Talbot, Edith Paxton, David Waddington, Elizabeth J Glass

**Affiliations:** 1 Division of Genetics & Genomics, Roslin Institute, Roslin, Midlothian, Edinburgh, EH25 9PS, UK; 2 ARK-Genomics Facility, Roslin Institute, Roslin, Midlothian, Edinburgh, EH25 9PS, UK

## Abstract

**Background:**

The response of macrophages to danger signals is an important early stage in the immune response. Our understanding of this complex event has been furthered by microarray analysis, which allows the simultaneous investigation of the expression of large numbers of genes. However, the microarray resources available to study these events in livestock animals are limited.

**Results:**

Here we report the development of a bovine macrophage specific (BoMP) cDNA microarray. The BoMP microarray contains 5026 sequence elements (printed in duplicate) and numerous controls. The majority of the clones incorporated on the microarray were derived from the BoMP cDNA library generated from bovine myeloid cells subjected to various stimuli, including over 900 sequences unique to the library. Additional clones representing immunologically important genes have been included on the BoMP microarray. The microarray was validated by investigating the response of bovine monocytes to stimulation with interferon-γ and lipopolysaccharide using amplified RNA. At 2 and 16 hours post stimulation 695 genes exhibited statistically significant differential expression, including; 26 sequences unique to the BoMP library, interleukin 6, prion protein and toll-like receptor 4.

**Conclusion:**

A 5 K cDNA microarray has been successfully developed to investigate gene expression in bovine myeloid cells. The BoMP microarray is available from the ARK-Genomics Centre for Functional Genomics in Farm Animals, UK.

## Background

Macrophages (mφ) play a key role in the immune system, acting as a bridge between the innate and adaptive immune responses. Mφ are sentinel cells that instigate the removal of invading pathogens, either by phagocytosis or by signalling to other components of the immune system. These signals include the release of various cytokines and the ability of mφ to act as antigen-presenting cells.

Mφ become activated when they interact with conserved, pathogen-specific stimuli, e.g. pathogen associated molecular patterns (PAMPs) such as lipopolysaccharide (LPS) and double-stranded RNA, characteristic of gram-negative bacterial or viral infections respectively. In addition cytokines, e.g. interferon-γ (IFN-γ) produced by T lymphocytes and other immune cells, activate mφ. Activation results in extensive remodelling of the mφ transcriptome, leading to morphological and physiological changes in the cell, including; alterations in shape, mobility and cell surface marker expression. These modifications equip the mφ to deal effectively with the invading threat. However, many pathogens have evolved the ability to circumvent the action of mφ and several have developed the ability to exist undetected within the mφ. These include a number of protozoan parasites including *Leishmania *species, *Toxoplasma gondii *and *Theileria annulata *[1,2].

The complexity of the mφ response to activation and infection is ideally suited for analysis by microarray technology, which allows the expression of thousands of genes to be investigated simultaneously. Numerous microarray experiments have now been published on the response of human and murine mφ to a range of stimuli, including; various pathogens, PAMPs and cytokines [reviewed in [3]]. In contrast, only a handful of experiments have been reported for livestock species. For example, five publications have investigated the response of bovine mφ and monocytes to stimuli [4-8]. Three of these studies have involved the use of commercially available human arrays. The most recent study used an in house bovine cDNA microarray [8], whilst the fifth study used a commercially available bovine array, which contains a limited number of immunologically important genes [6]. This partly results from an under-representation of immune genes in the EST datasets, caused by the generation of cDNA libraries from unactivated or uninfected immune tissue [9]. Although there are over 490,000 published bovine ESTs, IFN-γ and interleukin-2 (IL2) are only represented by one and two bovine ESTs respectively, whilst no bovine ESTs have been published that represent IL4. This problem is not restricted to livestock species, Wells and co-authors [10] have shown that mφ transcripts are poorly represented within the RIKEN mouse EST set, one of the most extensive collections of mouse transcripts currently available [11,12]. Eighteen percent of clones from a murine mφ cDNA library were novel, failing to match any published sequences [10]. Similarly, we have recently reported the construction of a bovine mφ (BoMP) specific cDNA library, in which over a third of clones appear to be novel, although this figure is decreasing as more bovine sequences become available [13]. The high percentage of novel sequences results from the inclusion of RNA samples in the library template that were collected from myeloid cells subjected to a range of different stimuli to maximize the number of transcripts associated with the immune response.

Here we report the use of BoMP library clones to construct a 5 K bovine macrophage specific (BoMP) cDNA microarray, which contains a large number of unique sequences. We also report the proof-of-principle experiment carried out using the BoMP microarray to investigate the response of bovine peripheral monocytes to LPS and IFN-γ stimulation, which stimulate cellular pathways of importance in the *in vivo *inflammatory and infectious response of myeloid cells.

## Results

### Characterization of the BoMP microarray

In total the BoMP microarray comprises 5026 clones printed in duplicate and numerous controls. Over 52% of clones match annotated gene sequences, a further 28% match ESTs and the remaining clones are currently unique to the BoMP library (Table 1 &Additional file 1). The majority of the clones were selected from the previously described BoMP cDNA library [13]. Approximately 10,000 clones from this library were considered for inclusion on the BoMP microarray. Clones that matched published sequences with E values less than e^-20 ^were included on the microarray, including those that only matched EST sequences (3599 clones). In addition, a representative clone from each cluster of sequences unique to the BoMP library was included on the microarray (220 clones). The remaining sequences were analysed using ESTScan [14,15], a web-based program that detects coding sequence by taking into account sequencing errors that frequently occur in single-pass sequences and can cause frame-shift mutations (174 clones). A further 592 unique BoMP clones were also randomly chosen for inclusion on the microarray.

T. *annulata *infected material was used to generate the BoMP library and as a result parasite sequence containing clones represent approximately 5% of the library [13]. Similarly, the infectious *T. annulata *sporozoites were prepared by homogenizing whole infected ticks, which has resulted in the presence of tick sequence containing clones in the library [13]. Eight clones known to contain non-bovine sequences have been included on the microarray; including C0006007g13 [EMBL: AJ817072] and C0006019k23 [EMBL: AJ820886], which match tick and parasite sequences respectively. These clones have been included as additional controls for a *T. annulata *infection experiment which will be reported elsewhere (Jensen *et al*., in prep.).

A number of immunologically important genes have not been detected in the BoMP library to date, partly because it has not been mined deeply. Therefore, a list of over 400 genes desirable for inclusion on the array was compiled. Where possible representative clones for these genes were selected for inclusion on the microarray from the various libraries housed at the ARK-Genomics facility (381 clones). The majority of these clones came from the Meat Animal Research Centre (MARC) and Beltsville Agricultural Research Centre (BARC) libraries MARC 1–4 BOV and BARC 5 BOV [16,17], e.g. macrophage migration inhibition factor (MIF) and integrin beta 6. Clones were not available for 60 genes on our compiled list. Therefore we generated amplicons for each of these clones using publicly available sequence information to design oligonucleotides suitable for amplification, e.g. IL6, caspase 3 (CASP3) and Toll-like receptor 9 (TLR9) (Table 2).

The BoMP microarray has been constructed at the ARK-Genomics Centre for Functional Genomics in Farm Animals [18] and submitted to ArrayExpress as ARK-Genomics Bovine Macrophage Specific-BoMP-cDNA microarray 5K v1.0 and has the accession number A-MEXP-495 [19,20].

### Analysis of microarray quality

Five randomly selected microarray slides from the print run were hybridized with Panomer 9 oligonucleotides (Molecular Probes). This hybridization visualized the size and shape of all printed spots, which allowed us to ensure uniformity in the amount of DNA spotted by each pin and to check the integrity of each spot. This test hybridization confirmed that the microarray printing had been successful (data not shown).

Normalized data from the experiment described below was used to investigate the consistency of the intensity measurements for replicate spots. The contributions of slide, gene, replicate spots and channel to the variability of the logarithm of the intensities were assessed by fitting them as random effects in a linear model. The fixed effects of animal and time were also included in the model. Intensity differences between genes were the largest contributor, accounting for 79% of the variation (Table 3, variance %). Almost all the remaining variation was accounted for equally between replicate spots and between channels, leaving little contribution from slides. Therefore, approximately 80% of the overall measured variation was of biological rather than technical origin. This summary is an oversimplification, which is illustrated by the variation observed in values when the time points are examined separately (Table 3, last 6 columns) and results from the failure to allow for the effects of differentially expressed genes in the 2 and 16 hour samples. The results for the 0 hour samples would be expected to be similar to that from a self-self hybridization and they do show more agreement that the other time points. The correlations between channel log(intensities) for the 0, 2 and 16 hour time points were 0.95, 0.89 and 0.88 respectively (calculated as (100-channel%)/100 from the last 3 columns of Table 3).

### Microarray analysis of monocyte activation

As a further evaluation of the BoMP microarray, it was used to investigate the response of bovine peripheral monocytes to stimulation. As part of a larger experiment, peripheral monocytes were isolated from six Holstein-Friesians under cold conditions before stimulation with LPS and IFN-γ. RNA was isolated from the cells of each animal at 0, 2 and 16 hours post activation. The quantity of total RNA (totRNA) isolated from these cells was too low for direct use in microarray experiments and therefore amplified RNA (aRNA) was generated.

The parameters used to identify genes exhibiting statistically significant differences in gene expression during the time course were a 2 fold or greater change in gene expression and a false discovery rate value (FDRmax) less than 0.01. Using these criteria 713 clones exhibited statistically significant changes in gene expression during the time course. Several of these clones represented the same gene and therefore a total of 695 genes exhibited differential expression (Additional file 2). Twenty-six of these were sequences unique to the BoMP library [13]. These included C0006011i04 [EMBL: AJ818203] that was up-regulated 8.4 fold by 16 hours post activation and C0005209g19 [EMBL: AJ816691] that was down-regulated 13.2 fold by 16 hours post activation. A further 171 sequences that exhibit differential expression currently only match ESTs. Putative gene names have been assigned to the remaining 498 sequences. The gene ontology information that is available for 398 of these genes illustrates the broad range of biological processes affected by activation with LPS and IFN-γ, including; signal transduction, transcription, immune response, apoptosis, cell proliferation, metabolism, intracellular transport, translation and proteolysis. Furthermore, 190 of the genes have been incorporated into KEGG pathways, with the largest number of genes being involved in cytokine-cytokine receptor interactions, haematopoietic cell lineage, TLR signalling, mitogen activated protein kinase (MAPK) signalling, Jak-STAT signalling, apoptosis, focal adhesion, complement and coagulation cascade, regulation of actin cytoskeleton and calcium signalling.

The limited number of time points sampled in this experiment restricts the information that can be obtained from cluster analysis. However, the 695 genes can be separated into groups exhibiting 8 broad expression patterns. The genes up-regulated at 2 hours fall into 3 groups; those that remain up-regulated (133 genes) including chemokine (C-C motif) ligand 2 (CCL2) and prion protein, those that return to resting levels (132 genes) including v-rel reticuloendotheliosis viral oncogene homolog (REL) and finally TIMP metallopeptidase inhibitor 1 (TIMP1) that is down-regulated beyond the resting state level. The genes down-regulated at 2 hour post activation can be grouped in a similar manner; those that remain down-regulated (48 genes) including v-fos FBJ murine osteosarcoma viral oncogene (FOS), those that return to resting levels (114 genes) including chemokine (C-C motif) receptor 2 (CCR2) and finally C0006007l14 [EMBL: AJ817952] which is up-regulated above the resting state level. The remaining genes exhibit a delayed alteration in expression that was observed only at 16 hours, and were either up-regulated (110 genes) e.g. fibronectin 1 (FN1) or down-regulated (151 genes) e.g. CD86 antigen.

Eighteen differentially expressed genes are represented by more than one clone. The duplicate clones of 13 of these genes are grouped together by expression pattern, e.g. FN1 and CCR1. The duplicate clones for the remaining 5 genes, e.g. IL12A, fall into different groups. Further analysis shows that the duplicate clones exhibit similar hybridization patterns, but the differential expression detected by one clone lies outside the threshold criteria used to identify differentially expressed genes.

#### 2 hours post LPS & IFN-γ stimulation

A total of 444 genes exhibited differential expression in monocytes at 2 hours post activation compared to resting monocytes (≥ 2 fold, FDRmax ≤ 0.01). Of these 279 were up-regulated and the top 25 genes exhibiting greatest up-regulation are listed in Table 4. Eight of these are chemokines, including IL8 and CCL2. Interestingly the prion protein was up-regulated on average 28.1 fold after 2 hours activation. Three of the most up-regulated genes are only represented by ESTs.

A further 165 genes are down-regulated 2 hours post activation compared to resting cells and the top 25 genes are listed in Table 5. These include IL16, CCR2 and MAPK14. Several of the most down-regulated genes are transcription factors, e.g. CCCTC-binding factor (CTCF), TAF7 RNA polymerase II, TATA box binding protein-associated factor (TAF7) and nuclear factor (erythroid-derived 2) (NFE2).

#### 16 hours post LPS & IFN-γ stimulation

A total of 457 genes exhibit differential expression in monocytes at 16 hours post activation compared to resting monocytes (≥ 2 fold, FDRmax ≤ 0.01). Of these 253 are up-regulated and the top 25 genes exhibiting greatest up-regulation are listed in Table 6. There is considerable overlap between the top 25 genes up-regulated at 2 and 16 hours post activation, including IL8 and prion protein. Other up-regulated genes include IL1β, IL2 receptor β chain (IL2RB) and actinin α1.

A further 204 genes are down-regulated at 16 hours post activation and the top 25 genes are listed in Table 7. These include the previously mentioned unique sequence C0005209g19 [EMBL: AJ816691] and five sequences that only match ESTs. Two ribonucleases are down-regulated, as well as cell surface receptors; IL6 receptor (IL6R), mannose receptor, C type 1-like 1 (MRC1L1) and CD59. Only 2 genes are found in the top 25 down-regulated genes at 2 and 16 hours post activation; IL16 and chromosome 6 open reading frame 32 (C6orf32) protein.

### Validation of microarray results

To validate the results from the microarray experiment, thirty genes were chosen for reverse transcription-polymerase chain reaction (RT-PCR) analysis. The mRNA levels of all thirty appeared to follow the same pattern as seen on the microarray (data not shown). The correct identification of the representative clones for these 30 genes was confirmed by sequence analysis (data not shown). Ten of these genes were selected for further quantitative RT-PCR (qRT-PCR) analysis to verify the abundance of mRNA present in the original totRNA samples from the six Holstein-Friesians used to generate the aRNA for the microarray experiment (Table 8). These genes were chosen because they displayed a range of differential expression during the time course. CCL2, prion protein and IL6 were highly up-regulated during the experiment, whilst FOS was down-regulated. FN1, CCR1 and complement component 1, r subcomponent (C1R) were up-regulated by 16 hours post activation, whilst CD86 was down-regulated by this time-point. CD9 and CD44 did not differ significantly during the time course.

**Figure 1:**
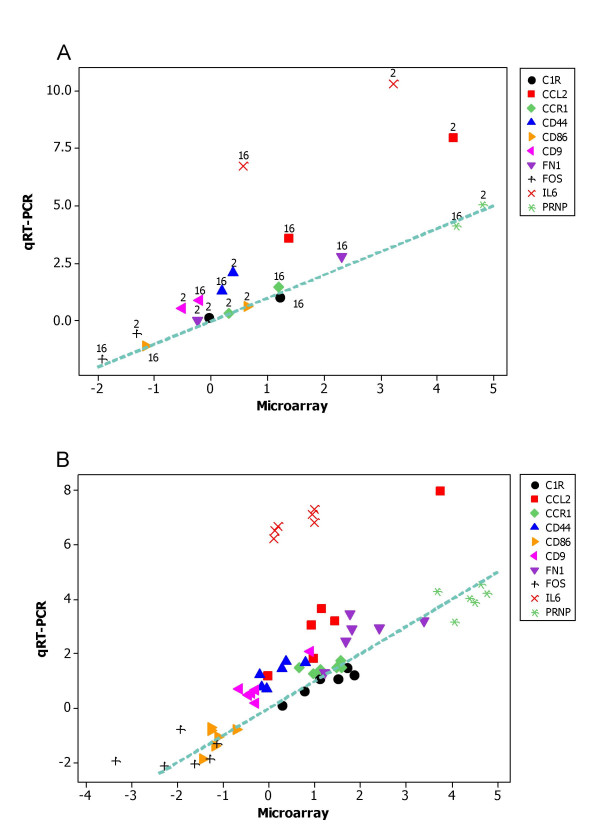
**Scatterplots comparing the differential expression measured by qRT-PCR and microarray analysis for 10 investigated genes at 2 time points after activation**. A, average log(differential expression). The numbers indicate the time post activation. B, log(differential expression) of individual animals at 16 hours post activation. The 10 investigated genes were C1R (black circle), CCL2 (red square), CCR1 (green diamond), CD44 (blue triangle), CD86 (orange triangle), CD9 (pink triangle), FN1 (purple triangle), FOS (black cross), IL6 (red cross) and PRNP (green star). The dotted lines denote the lines of equality (y = x).

The correspondence between the qRT-PCR and microarray expression measurements is shown in Figure 1. There is good agreement between the average log(fold changes) for the 10 genes, with an overall correlation of 0.78 (Figure 1A). The means for 6 genes; C1R, CCR1, CD86, FN1, FOS & PRNP, lie close to the line of equality. CD9 and CD44 exhibit 2–3 times higher fold changes by qRT-PCR than by microarray analysis and CCL2 results are 5 and 13 times greater. The fold change was dramatically different for IL6, being 70 and 130 times greater by qRT-PCR analysis than microarray analysis. The correlation between the qRT-PCR and microarray data was further investigated by comparing the results for individual animals. The individual animal values for each gene at 2 hours (data not shown) and 16 hours (Figure 1B) post activation cluster together for 9 of the 10 genes. The CCL2 values have the most spread and one outlying animal influences the difference between the average microarray and qRT-PCR results (Figure 1A). However, the results for IL6 cluster together, illustrating the consistency of the disparity between qRT-PCR and microarray results for this transcript. Investigation of the raw data failed to identify any obvious reason for the lower fold changes measured by microarray analysis, in particular the disparity could not be explained by saturation effects nor excessively low channel intensities (data not shown).

## Discussion

Microarray analysis provides an ideal methodology to investigate the gene expression profile of thousands of genes simultaneously, which allows a broader understanding of complex events, such as mφ activation and differentiation. Here we describe the construction of a bovine macrophage specific cDNA microarray. The majority of the 5026 clones present on the BoMP microarray have been selected from a normalized bovine mφ specific cDNA library [13]. The library was generated from *Bos taurus *and *B. indicus *derived monocytes and mφ subjected to various stimuli, including infection with the protozoan parasite *T. annulata*, to maximize the number of transcripts present in the library. A normalized rather than a subtracted library was generated to ensure that non-mφ specific genes were represented, as we were interested in the complete picture of events during host-mφ activation and not just the subset unique to mφ. Furthermore, the majority of murine mφ genes exhibiting differential expression after LPS stimulation were not mφ specific [10]. Therefore all sequenced clones that matched published sequences were included on the BoMP microarray. The BoMP library also contains a large number of unique sequences and a proportion of these were also included on the microarray. Additionally, a further nearly 450 genes were selected for inclusion on the microarray. Therefore the BoMP microarray is focussed towards investigating the response of myeloid cells, but is of sufficient size and diversity to allow for successful normalization of microarray data.

The BoMP microarray has been validated by investigating the response of bovine monocytes to IFN-γ and LPS activation. These compounds are potent, natural activators of myeloid cells and are frequently used, individually or together, to experimentally stimulate monocytes [reviewed in [3]]. A common reference design was used for this microarray experiment. Because only three time-points, or treatments, were investigated it would have been straightforward to use a loop design, which allows the direct comparison of treatments resulting in higher statistical power than can be obtained from a common reference design [21]. However, the experiment described here forms part of a larger experiment investigating variation in the response of bovine monocytes derived from Holstein-Friesians (*B. taurus*) and Sahiwal (*B. indicus*) cattle. Therefore a common reference design was used to allow multifactorial analyses to be carried out looking across the time course and between cattle breeds. In addition, the same common reference sample has been used in a separate microarray experiment investigating the response of bovine monocytes to *T. annulata *infection (Jensen *et al*., in prep).

There are an increasing number of microarrays available to investigate bovine gene expression [reviewed by [3]]. A sub-set of these have been designed to specifically investigate the immune response. The majority of publications have made use of versions of the bovine total leukocyte (BoTL) microarray [22]. The latest version, BoTL-5 contains 1391 genes and controls [23]. Other arrays include the bovine immune-endocrine cDNA microarray containing amplicons representing 167 genes [24]. Recently a bovine innate immunity microarray has been described which contains over 6800 clones [25]. The majority of these clones have been selected from normalized and subtracted libraries and have not been sequenced. Therefore there is likely to be considerable redundancy on this microarray and its full scope is not known.

However, microarray studies investigating the response of bovine monocytes and mφ to infection and activation have not made use of these focussed microarrays. The response of monocytes to *Escherichia coli *0157:H7 LPS was investigated using Incyte Genomics UniGEM V 1.0 human cDNA microarray [4]. Cells were activated for 3 hours and bovine monocyte RNA hybridized with approximately 80% of the gene targets. A total of 44 differentially expressed genes were identified, 21 of which are represented on the BoMP microarray. However, only 13 of these genes exhibited differential expression in the study reported here, including pentraxin-related gene, rapidly induced by IL1β (PTX3), CASP8 and FADD-like apoptosis regulator (CFLAR) and TIMP3. The remaining 8 genes identified in the earlier study as being differentially expressed during activation are not exhibiting differential expression during the time course reported here, e.g. CASP4 and tumour necrosis factor (TNF-α). These results could indicate a genuine difference in the response to the two different stimuli, be due to other technical differences between the two studies or be due to the failure of the DNA from the representative clone to hybridize correctly. The latter is true for TNF-α, the expression of which we have previously shown to differ during monocyte activation (data not shown). The failure of this clone has prompted us to include additional clones representing TNF-α on the next version of the microarray, which is currently under construction.

There is a considerable difference in the number of differentially expressed genes identified in LPS stimulated cells (44 genes) compared to the study reported here (695 genes). The earlier study compared gene expression in LPS stimulated cells with cells cultured in medium only and therefore the response of the cells to LPS alone was investigated [4]. In contrast, the study reported here has investigated the response of cells to LPS and IFN-γ compared to resting cells and therefore the cells are also responding to being in culture conditions for the first time. Furthermore, our cells were stimulated immediately after isolation, whilst the previous study used cells that were cultured overnight before treatment [4].

Bovine derived microarrays have been used to investigate gene expression in bovine monocytes and mφ in two studies [[6] &[8]]. The response to alveolar mφ to infection with virulent and attenuated strains of *Mycobacterium bovis *has been investigated using an in house cDNA microarray [8]. In addition, a commercially available bovine microarray produced by Pyxis Genomics [26] was used to compare gene expression of bovine monocyte-derived mφ (MDM) infected with *M. avium *subspecies *paratuberculosis *and *M. a. avium *[6]. This cDNA microarray was constructed from bovine spleen and placental cDNA libraries; however, immunologically important genes are poorly represented on the microarray. For example, the principal proinflammatory cytokines, IL1β, IL6 and TNF-α, are not represented. Therefore the microarray is of limited use when investigating aspects of the immune system.

Microarray analysis has been used to investigate the response of human and murine derived mφ to LPS and IFN-γ [reviewed in [3]]. A total of 219 genes exhibited differential expression 6 hours post LPS treatment in the murine cell line RAW264.7 [27]. Of these 85 (38.8%) are represented on the BoMP microarray and 42.4% (36) of these exhibit similar fold changes. The mφ response to a range of pathogens and PAMPs has been investigated simultaneously [28,29]. A shared activation program of expression changes in 191 human mφ genes was found in response to a range of bacterial pathogens, the majority of which were also activated by LPS [28]. Ninety-four of these genes are represented on the BoMP microarray and 64% of up-regulated genes in the activation program were also up-regulated in the current study, e.g. ninjurin 1 (NINJ1), prostaglandin-endoperoxide synthase 2 (PTGS2), nuclear factor of kappa light polypeptide gene enhancer in B-cells inhibitor, alpha (NFKBIA) and jagged 1 (JAG1).

The expression of 10 genes was investigated by qRT-PCR to validate the microarray results. The same RNA samples were used for this analysis, rather than the collection of additional samples, because the principal aim was to confirm to results obtained from the microarray experiment rather than to investigate the expression of particular genes. Overall there was good correlation between the microarray and qRT-PCR results, with a correlation value of 0.78. This level of correlation is comparable to that seen in a large scale validation study of over 1300 genes recently reported [30]. However, the dominant pattern observed in the comparison of microarray and qRT-PCR results reported here closely follows the line of equality, which was not observed in the large scale study [30]. The average logarithms of differential expression for the two time points were remarkably similar for 6 of the 10 genes. The qRT-PCR analysis was more sensitive for the remaining 4 genes and detected increased differential expression over the microarray analysis, particularly for IL6, which was underestimated by as much as 100 fold. There are several possible explanations for the disparity between these methodologies, including; fluorescence saturation and specificity. However, analysis has confirmed that the disparity in IL6 results is not caused by the saturation of the fluorescence on the microarray (data not shown). Closely related gene-family members, sharing more than 80% sequence homology, can cross-hybridize in cDNA microarray experiments resulting in false positive results [31]. However, this can not explain the results for IL6, which is not part of a closely related gene family and the hybridization signals were lower than expected. The most probable cause of the disparity in IL6 results is sub-optimal hybridization of IL6 on the microarray. In contrast, the qRT-PCR amplification is optimized on a gene basis.

Due to the limited amount of totRNA generated from the peripheral monocytes, the microarray experiment was carried out using aRNA. The suitability of this approach has been validated by previous studies [32]. The qRT-PCR was carried out on the original totRNA samples used to generate the aRNA used in the microarray experiment. The similarity between the majority of the results supports the use of aRNA for microarray analysis. However, variable amplification of transcripts may account for the disparity between the qRT-PCR and microarray results for IL6. This could be investigated by repeating the qRT-PCR analysis using the aRNA samples. Unfortunately, this was not possible due to limited amounts of the samples remaining. The log(fold changes) were encouragingly consistent between animals for all but one of the genes examined. A comparison of the results of statistical significance tests for the two methodologies has not been attempted. The microarray results would require recalculating for a fair comparison, as they are penalized for the multiple testing of over 5000 genes and also have the residual variances for each gene "shrunk" towards the median residual variance to improve the false discovery rate for the list of top genes exhibiting differential expression.

Further investigation of the validity of the microarray results concentrated on the top gene lists (Tables 4, 5, 6, 7), by *in silico *analysis, searching the literature for corroborative data. Unfortunately, corroborative data has not been found for a proportion of the genes. Several of the most differentially expressed genes currently only match ESTs or are unique to the BoMP library. Exploration of these may provide insights into novel pathways involved in the reprogramming of the innate immune cell after infection or inflammation. Further work on the bovine genome sequencing project should provide more information on these sequences in the future.

Results for two of the top differentially expressed genes are inconsistent with previously published data. Dihydropyrimidinase-like 2 (DPYSL2) is down-regulated over 5 fold in this study. However, expression of the gene has been reported as up-regulated upon monocyte activation [33]. Similarly, previous studies have shown that C1Q is up-regulated during LPS and IFN-γ stimulation [34]. In the current study both C1QA and C1QB are down-regulated 4.8 fold and 2.9 fold respectively. The disparity between the results may result from differences in experimental design, cell types or species differences, or from other technical differences between the studies.

Many of the top differentially expressed genes have been shown to be differentially expressed in activated myeloid cells by previously microarray analysis. Sixteen of the genes were listed in the mφ activation program, including; N-sulfoglucosamine sulfohydrolase, adrenomedullin and ribonuclease k6 [28]. Similarly seven of the top genes were also described in the study of gene expression in RAW264.7 cells, e.g. exportin 1 [27] and three of the top genes were differentially expressed in the only other published paper on bovine monocyte gene expression, e.g. TNF-α induced protein 3 (TNFAIP3) [4].

The biological function of prion protein is still unclear, although mRNA and protein levels increase upon activation of many leukocytes, including T lymphocytes [35]. Mφ from prion protein knockout mice show lower rates of phagocytosis and recruit a different subset of leukocytes, suggesting that the protein plays an important role in mφ function [36]. Differentiation of monocytes to dendritic cells (DC) increases surface expression of prion protein [37] and IFN-γ stimulation triggers increased prion protein expression in CD14+ peripheral cells [35].

Exposure to IFN-γ also induces phorbol-12-myristate-13-acetate-induced protein 1 (PMAIP1) expression, which disrupts the mitochondrial outer membrane integrity and is involved in apoptosis [38]. Apolipoprotein B mRNA editing enzyme, catalytic polypeptide-like 3A (APOBEC3A) acts to inhibit retroviral replication and expression of the closely related proteins APOBEC3B and APOBEC3C are stimulated by IFN-γ [39].

An additional 13 genes from the top differentially expressed genes have been shown to be differentially expressed in myeloid cells after LPS treatment. These include up-regulation of IL2RB [40] and down-regulation of IL6R [41], CCR2 [42] and CD59 [43]. Similarly, the nuclear receptor subfamily 4, group A, member 3 (NR4A3), which is mediated by NF-κB signalling is up-regulated in RAW264.7 stimulated with LPS [44]. Furthermore, the zinc transporter solute carrier family 39 member 8 (SLC39A8) is expressed at very low levels in unstimulated monocytes, but levels increase after LPS stimulation [45].

The information described in this report, particularly the expression changes in unique or unknown genes, may in the future suggest new candidates for disease resistance genes. Furthermore, the results may suggest new ways to effectively stimulate the innate immune system, leading to improvements in the design of vaccines and adjuvants.

## Conclusion

Here we report the construction of a 5 K bovine macrophage specific cDNA microarray, which has been validated by investigating the response of bovine monocytes to culturing and stimulation with IFN-γ and LPS. Over 690 differentially expressed genes were identified and the results for a selection of these genes have been confirmed by qRT-PCR analysis. Therefore the BoMP microarray is a useful resource for investigating gene expression in bovine myeloid cells. A second version of the microarray is currently under construction, which contains an additional 500 clones to improve the representation of various biological pathways on the microarray. Both versions of the BoMP microarray will be publicly available through the ARK-Genomics Centre for Functional Genomics in Farm Animals [18].

## Methods

### Construction of the BoMP microarray

#### Clone selection

5026 sequence elements have been chosen for inclusion on the BoMP microarray, see Additional file 1 for a complete annotated list. These have been derived from a number of resources (Table 1). The majority of the clones (4585 clones) were derived from the normalized BoMP cDNA library [13], generated from RNA samples collected from *B. taurus *and *B. indicus *derived cells subjected to various stimuli. A further 381 clones were selected from the MARC 1–4 BOV libraries and others available at the ARK-Genomics facility [16,17]. In addition, 60 amplicons were generated to represent immunologically important genes that were not available in the libraries at our disposal, e.g. IL6 and IL8.

#### Generation of amplicons

Sixty immunologically important genes were selected for amplicon generation (Table 2). Oligonucleotides were designed for the 60 genes using the Primer3 website [46,47]. First strand cDNA was reverse transcribed from 0.5 μg myeloid cell totRNA using oligo(dT) primer and Superscript II (Invitrogen) according to the manufacturer's instructions. Two microlitres of the cDNA was amplified using the gene specific primers in a 100 μl polymerase chain reaction (PCR) containing the following; 1 × PCR buffer, 1.5 mM MgCl_2_, 200 μM dNTP mix, 200 nM forward and reverse primers and 0.5 units Taq polymerase (ABgene). PCR amplification was carried out using the following cycle profile: one cycle of 95°C for 3 minutes, 60°C for 30 seconds and 72°C for 45 seconds, followed by 39 cycles of 95°C for 30 seconds, 60°C for 30 seconds and 72°C for 45 seconds. The final cycle had an extension at 72°C for 5 minutes. The PCR products were purified using QIAquick Gel Extraction Kit (Qiagen) before being cloned into pGEM-T easy vector (Promega) according to the manufacturer's instructions. The resulting plasmids were sequenced to ensure amplification of the specific product (data not shown).

### Microarray construction

The BoMP microarray was constructed from the 5026 clones described above and additional controls, including; chicken and bovine genomic DNA, calf thymus DNA, salmon sperm DNA, glyceraldehyde-3-phosphate dehydrogenase (GAPDH), γ-actin, landing lights, spotting buffer and the Alien SpotReports 1–10 (Stratagene). The controls are distributed evenly across the microarray. The plasmid DNA was purified using MagAttract 96 Miniprep chemistry on a Biorobot 8000 platform (Qiagen). The cDNA inserts were PCR amplified using the oligonucleotides CGATTAAGTTGGGTAACGC and CAATTTCACACAGGAAACAG in 50 μl reactions using 1 μl plasmid template. Amplified DNA was purified by Multiscreen 384 well PCR purification plates (Millipore) on a Multiprobe II liquid handling platform (Perkin Elmer). The presence of products was confirmed by agarose gel electrophoresis and quantified by Picogreen assay (Molecular Probes) on a Flouroskan Ascent flourescent plate reader (Thermo Life Science).

DNA was resuspended to 150 ng/μl in spot buffer (150 mM sodium phosphate, 0.01% sodium dodecyl sulphate) before being spotted in duplicate on to amino-silane coated GAPSII slides (Corning) using a Biorobotics MicroGrid II spotter (Genomic Solutions). The microarray is laid out in a 12 × 4 grid, where each block corresponds to one of 48 print tips. Each block comprises a 16 × 15 grid of spots.

Printed microarray slides were treated using succinic anhydride and 1-methyl-2-pyrrolidinone (Sigma) to block unbound amino groups, followed by a wash in 95°C MilliQ water before hybridization.

### Sample collection

Peripheral blood was isolated from 6 Holstein-Friesian (*B. taurus*) cattle maintained at the Roslin Institute, UK. These animals, 2 females and 4 males, were between 1–3 years of age and kept on pasture. The blood was collected aseptically into acid citrate dextrose and immediately stored on ice. Under cold conditions peripheral blood mononuclear cells (PBMC) were separated by density gradient centrifugation on Lymphoprep (Axis-Shield), washed three times with phosphate buffered saline (PBS) and resuspended at 10^7 ^cells/ml in PBS supplemented with 1% foetal bovine serum (FBS). Peripheral monocytes were isolated from PBMC by positive selection using a predetermined optimum concentration of the monoclonal antibody IL-A24, which recognizes signal-regulatory protein alpha (SIRPA) [48], and the MACS system according to the manufacturer's instructions (Miltenyi Biotec). FACS analysis confirmed that the cell purity exceeded 95% (data not shown).

The purified monocytes from each animal were resuspended at 10^7^cells/ml in RPMI-1640 medium supplemented with 10% FBS, 20 ng/ml LPS derived from *E. coli *serotype 055:B5 (Sigma) and 300 U/ml recombinant ovine IFN-γ, kindly supplied by Dr. G. Entrican (Moredun Research Institute). Cells from each animal were divided into 3 aliquots and incubated at 37°C in a 5% CO_2 _incubator. The cells were harvested at 0, 2, and 16 hours post activation. The activated monocytes were pelleted, washed with PBS and stored in RNAlater (Ambion) at 4°C before RNA extraction.

### RNA preparation

totRNA was extracted from the 18 cell samples using the RNeasy mini kit (Qiagen) according to the manufacturer's instructions. The quality and quantity of the resulting RNA was determined by gel electrophoresis, spectrophotometry at 260 nm and 280 nm and by Agilent 2100 Bioanalyzer. Linearly amplified aRNA was generated from 0.5 μg totRNA using the MessageAmp aRNA kit (Ambion) according to the manufacturer's instructions and incorporating modified 5-(3-aminoallyl)-UTP. All totRNA samples underwent a single round of amplification. The quality and the quantity of the resulting aRNA was analysed by spectrophotometry and Agilent 2100 Bioanalyzer.

### RNA labelling & hybridizations

Fluorescent cyanine (Cy) dyes, either Cy3 or Cy5 as appropriate, were indirectly incorporated into each 1.2 μg aRNA sample by coupling to the modified UTP using the protocol described on the ARK-Genomics website [18]. The labelled aRNA was purified using a DyeEx spin column (Qiagen) and the labelling efficiency was determined by running 0.5 μl of each sample on a 1% agarose gel.

Hybridizations were carried out in a GeneTac automated hybridization station (Genomic Solutions). The Cy3 and Cy5 labelled aRNA were mixed and added to 125 μl hybridization solution (ARK-Genomics protocols [18]) and hybridized onto the BoMP microarray for 12 hours. The microarray slides were then washed in wash buffers of increasing stringency (Genomic Solutions). After removal from the hybridization station the microarray slides were sequentially washed in post wash buffer and isopropanol for one minute before being dried by centrifugation at 220 g for 6 minutes. The dried slides were scanned in a Scanarray 5000 XL scanner (GSI Lumonics) at constant laser power of 80% and 78% for Cy3 and Cy5 respectively.

### Experimental design

The experiment described here was part of a larger microarray experiment of 36 microarray slides comparing the response of monocytes derived from 6 Holstein-Friesian (*B. taurus*) and 5 Sahiwal (*B. indicus*) cattle to stimulation with IFN-γ and LPS, which will be described elsewhere (Jensen *et al*., in prep). The RNA samples from each animal and time point were analyzed separately. The resulting aRNA from each sample was hybridized in competition with a pooled reference sample, made up of 5 resting monocyte aRNA samples; 3 Sahiwal and 2 Holstein samples. A common reference design was used to simplify the multifactorial analysis of the complete experiment. The reference sample was labelled with Cy3 and the treatment sample with Cy5 on each microarray slide. The microarray data has been submitted to ArrayExpress and assigned the accession number E-MAXD-16 [19,20].

### Microarray data analysis

**Figure 2:**
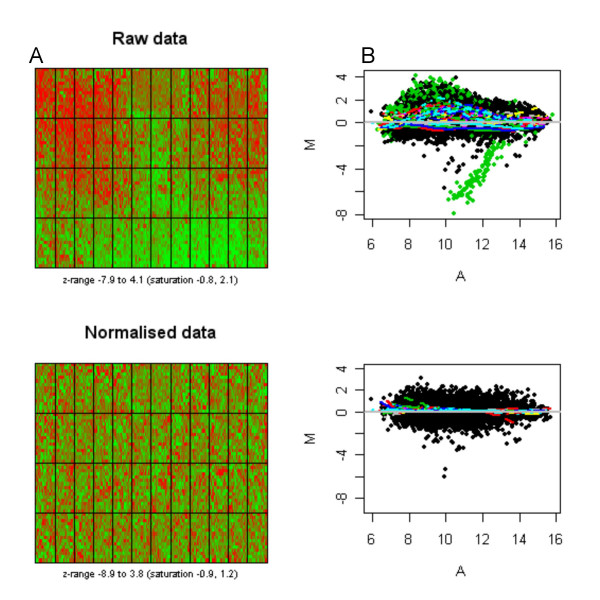
**Representative spatial heat maps and M-A plots of raw and normalized data**. A, spatial heat map for a representative microarray slide before and after normalization. The footnote gives the range of M values for the slide (z-range) and the range excluding the extreme 5% tails of the distribution of M values (saturation) which are excluded from the plot. B, M-A plots for the same representative microarray slide before and after normalization. Green spots denote control genes and the coloured lines represent the curve for each print tip.

Microarray spot intensity and quality data were extracted from the scanned images using the BlueFuse software version II (BlueGnome). Each slide was normalized separately, using the log_2_-ratios of treatment to reference intensities for all the non-control spots. Normalization and analysis was based on the Limma package of Bioconductor [49], with additional plotting from the Bioconductor Marray package. However, the Limma models were modified at both the normalization and analysis stages. The normalization was a 2-step process of spatial then intensity dependent bias correction. The spatial bias correction was carried out separately for each 2 × 2 group of blocks, by subtracting corresponding row and column means (RC correction, excluding control spots) from each data spot. This simple correction has been used in crop experiments to remove spatial trends and has also been suggested for similar trends across microarrays [50]. The rows and columns may stretch across the whole slide (global) or only across the spots within a block, to more strongly reflect local spatial patterns. The choice of 2 × 2 groups of blocks results in a smoothing of the spatial pattern intermediate between these two. The intensity dependent bias was removed by local block-lowess [51]. The choice of level, global or local, for the two normalization steps was informed by examination of spatial heat diagrams and M-A plots for all possible normalization combinations (Figure 2).

The time course effects of the log ratios of treatment to reference for means of replicate spots on each microarray slide were analyzed by regression models, which allowed for repeated observations from each animal. The Limma eBayes correction [52] was used to shrink the residual variances of genes towards their approximate median value. The effect of time was assessed by two comparisons; firstly, the values from resting samples were compared to activated sample values (at both 2 or 16 hours). Secondly, the 2 hour values were compared to the 16 hour values. Comparisons were made using Smyth's moderated t-test [52]. FDRmax values for each contrast were calculated using the method of Benjamini and Hochberg [53], the default multiple testing method in Limma. Genes with FDRmax values less than or equal to 0.01 were considered as significantly different. To refine the list of differentially expressed genes further to those of probable biological relevance, only those genes exhibiting a 2 fold or greater average change at 2 hours or 16 hours post activation compared to resting cells were considered.

### Validation of microarray results

Quantitative RT-PCR was carried out on 10 genes to verify the microarray results. Oligonucleotides were designed for each gene using Primer3 [46,47] and Netprimer (Biosoft International) software [54] (Table 8). The qRT-PCR analysis was carried out on the same totRNA samples from Holstein-derived monocytes used to generate aRNA for the microarray experiment. First strand cDNA was reverse transcribed from 0.5 μg totRNA using oligo(dT) primer and Superscript II (Invitrogen) according to the manufacturer's instructions. The resulting cDNA was diluted 1:50 for all genes except FOS, when the cDNA was diluted 1:10. FOS was down-regulated during activation and therefore a more concentrated cDNA sample was required for successful qRT-PCR amplification of FOS in the 16 hour time points.

The mRNA levels of each transcript were quantified by PCR using the Platinum SYBR Green qPCR Supermix UDG kit (Invitrogen). Reactions were carried out in 20 μl volumes containing; 1 × supermix (Sybr Green, Platinum taq DNA polymerase, dNTPs, UDG and stabilizers), 0.4 μl Rox dye, 1 μl forward and reverse primers at predetermined optimal concentrations and 5 μl diluted cDNA. Amplification and detection of products was carried out using a Mx3000P PCR machine (Stratagene) with the following cycle profile: 50°C for 2 minutes, 95°C for 2 minutes followed by 40 cycles of 95°C for 15 seconds and 60°C for 30 seconds. The detection of a single product was verified by dissociation curve analysis. Each PCR experiment was carried out in triplicate and contained several non-template controls and a log_10 _dilution series of the representative clone from the array or activated monocyte RNA. The relative quantities of mRNA were calculated using the method described by Pfaffl [55]. The qRT-PCR results for Chromosome 13 open reading frame 8 (C13orf8) [EMBL: AJ817183] were used to calculate differences in the template RNA levels and thereby standardize the results for the genes of interest. C13orf8 was selected from microarray and qRT-PCR analyses as a constitutively and moderately expressed gene in activated, *T. annulata*-infected and resting Holstein and Sahiwal derived monocytes (data not shown). Where Pfaffl [55] uses ratios of the control gene to target gene values, we use the differences between the corresponding logarithms of these values. The qRT-PCR values are then comparable with the log(intensity ratios) from the microarray analysis, both in scale and as estimates of the log(geometric mean) values.

## Authors' contributions

KJ designed the microarray, collected the RNA samples and drafted the manuscript. RT constructed the microarray and carried out the microarray experiments. DW statistically analyzed the microarray data. EP and KJ carried out the qRT-PCR analysis. EJG was involved in the microarray and experimental design. All authors read and approved the final manuscript.

## Supplementary Material

Additional File 1 **Annotation of the BoMP microarray**. An excel file of the annotation of the BoMP microarray is available as supplementary material and is also found on the Roslin Institute website [56]. Where possible the best human RefSeq hit has been listed for analysis purposes. Clones highlighted in blue denote those containing non-bovine sequences. Click here for file

Additional File 2 **List of clones exhibiting statistically significant differential expression during monocyte activation with IFN-γ and LPS**. An excel file listing the 695 genes exhibiting differential expression during monocyte activation (≥ 2 fold, P < 0.01, FDRmax < 0.01). Fold increases and decreases are highlighted in orange and green respectively. Genes shown in bold are represented by more than one clone. The calculated P values and FDRmax values are included for the 2 analyses of the affect of time; Pre v Post, resting sample compared to activated samples; 2 hr v 16 hr, 2 hour values compared to 16 hour values. Click here for file

## Figures and Tables

**Table 1:** Composition of the BoMP cDNA microarray.

**BoMP cDNA library clones**	
BLASTN hits	2185 (43.5%)
EST hits – bovine	1139 (22.7%)
human	156 (3.1%)
other	119 (2.4%)
Unique clusters	220 (4.4%)
ESTscans	174 (3.5%)
Unique singletons	592 (11.8%)
**Additional clones**	
MARC1-4/BARC5	355 (7.1%)
Other libraries	26 (0.5%)
Amplicons	60 (1.2%)
**Total**	**5026**

The percentage of clones included on the BoMP microarray from various sources is listed along with the annotation available for the BoMP library clones.

**Table 2:** Amplicons generated to represent 60 immunologically important genes.

Gene	Symbol	Accession No.	Size (bp)	Forward Primer 5'-3' sequence	Reverse Primer 5'-3' sequence
Annexin-A5	ANXA5	CO883855	700	ATG GCA CAG GTT CTC AGA GG	CCA AAT TGC CAG AAG TCT CC
Baculoviral IAP-repeat containing 5	BIRC5	BE486047	451	CCT GGC AGC TCT ACC TCA AG	CCC TGG AAA CGA CGT GTC TG
B-cell CLL/lymphoma 3	BCL3	CK771977	537	CTT CTT GGC ACT CGG TGT TC	GAC CAG AGG CAA TTT ACT ACC
Capping protein (actin filament) gelsolin like	CAPG	AY219899	615	TAT GGA GGG TGG AGA AGC TG	TGG GAC CCA AGA CCT GTA TC
Casein kinase II α1	CSNK2A1	X54962	405	GTT AAT ACA CAC AGA CCC CGA	GCT ATG GCA GTA ATC AAG GGC C
Caspase 3	CASP3	CK958902	679	GTG GAT GCA GCA AAC CTC AG	CCA TTT GCT CAC TTG GCA TAC
Caspase 7	CASP7	CK436554	549	TCT TCC AAA GTC ACC ACC AG	GGA GTG GCT CTT GGT TTG TG
Caspase recruitment domain family member 4	CARD4	CO886987	630	ATG TCG GAG CCA GGT ACA TC	GAA GTC CCA CCT CAG CAG AC
CD2 antigen	CD2	CN789613	544	CAG AAC TGT GAG CTG CAA GG	TTG CTG GTG AAC TTG TGT GC
CD3 antigen γ	CD3G	CD445323	518	GAA GGA GTT CGC CAG TCA AG	TGA TGA GAA TAG CCA ATA AGC AC
CD40 ligand	CD40LG	Z48469	652	TAA CGC AGC ATG ATC GAA AC	AGA ACT GTG GGT GTT TGC AG
CD63 antigen	CD63	AJ012589	606	TTC TCC TGC TGG TCT TCT GC	GGA TCT CCA CAA AGG CAA TG
CD80 antigen	CD80	BF039039	522	GGT ACT CCA ACC AGC GTC TC	AGG TGT AGG TGC CAC TGT CC
CD86 antigen	CD86	AJ291475	674	GCC TTC TTC AAC GAG ACT GG	GCA ATC CAG AGG ATG TGG TC
CD200 receptor 1	CD200R1	CK831048	552	CTG TGG ACA GAA AGC AGA GC	GGT TCT GAC ACC TTT ATC CAG
Chemokine (C motif) ligand 1	XCL1	AF354057	411	TCA GCC TCT TAC TGC ACA GC	AAA TGA GGT GGC TGG CTA GG
Chemokine (C-C motif) ligand 8	CCL8	S67954	667	TGA GAC CAA CTC AGC AAT CG	AGT GGA AGG ACC TCA CCA TC
Colony stimulating factor 2	CSF2	U22385	602	GAG GAT GTG GCT GCA GAA CC	CCT GTA TCA GGG TCA ACA TGG
Colony stimulating factor 2 receptor α	CSF2RA	BE480577	455	CCA AAT GCC AGC CTA AAT GTG	CGA GAG GTC TTG GAG GTG AC
Complement component 5 receptor 1	C5R1	X65861	396	ATC ATC CAG CAC AAC CAC TG	GCA GGA GGA AGG TGT AAC AG
FcγRIα (CD64)	FCGR1A	AF162866	630	TCT GGT CAA CCT GAG CTG TG	TAA GAT GCC AAG GGA GTT GC
FcγRIIIb (CD16b)	FCGR3B	AF132036	502	CTT TCA GCT GAC ACG CAA AC	TTT CCT GCT GCT CCC AGA G
Inhibitor of kappa light polypeptide gene enhancer in B-cells, kinase β	IKBKB	AJ414556	688	TTT GAC AAC AGC AGG GTC AC	TGC AGA CCA CGG TCT TAC TG
Inhibitor of kappa light polypeptide gene enhancer in B-cells, kinase γ	IKBKG	AJ414557	694	ATG GAG TGC ATC GAG TAG GG	AGA GAG GAG CTC ACC TTG TG
Insulin-like growth factor 1	IGF1	X15726	661	GTG ATC TGA GGA GGC TGG AG	AGG TGA TGG AGA AGG GAG TG
Integrin αV (CD51)	ITGAV	BM431105	505	CAT ATC TGC GGG ATG AAT CTG	TCC ATC TCT GAT TGC TGG TG
Integrin α6 (CD49f)	ITGA6	CK970520	554	TAC CCT GAT GTT GCT GTT GG	ATG TTG TCG TCT CCA CAT CC
Integrin β1 (CD29)	ITGB1	AF468058	691	TGT GTT TCA CTT CGC TGG AG	TTC GCT GTT CAC CTC ATC TG
Integrin β3 (CD61)	ITGB3	CN791459	530	TGT TCT CCA GCT CAT TGT GG	TCT TGC CAA AGT CAC TGC TG
Intercellular adhesion molecule (CD50)	ICAM3	L41844	628	CCA AGA GAT TGT CTG CAA CG	GCC CAA GAT CGC TAA CAC TC
Interferon α	IFNA	Z46508	439	GGT CCT GAT GCT CCT GAG AC	CCT GCA AGT TTG TTG AGG AAG
Interferon β1	IFNB1	M15477	568	TCT CCA CCA CAG CTC TTT CC	TCT CAC CTC AAA GTC ACA TTG
Interferon γ	IFNG	M39867	561	GGA GCT ACC GAT TTC AAC TAC TCC G	GCA GGC AGG AGG ACC ATT ACG
Interferon regulatory factor 2	IRF2	AJ490936	686	AAA CTG GGC CAT CCA TAC AG	TGT TGG ACG TGA CAA AGG TG
Interleukin 1β	IL1B	M37211	699	AAG GCT CTC CAC CTC CTC TC	TGG TTG CTC ATC AGA AGC TG
Interleukin 1 receptor-associated kinase 1	IRAK1	BG692698	405	CTC TCT CCT GGC CTC AGC	CTT CCG CCT CCT CTT CAA C
Interleukin 2	IL2	M13204	498	GGT CGT TCA TGT CAG CAA TG	GAG GCA CTT AGT GAT CAA GTC
Interleukin 4	IL4	M77120	451	GCA TTG TTA GCG TCT CCT GGT AAA C	CTT CAT AAT CGT CTT TAG CCT TTC C
Interleukin 6	IL6	X57317	620	ATG AAC TCC CGC TTC ACA AGC	TAC TTC ATC CGA ATA GCT CTC
Interleukin 8	IL8	AF232704	660	CAG CAG AGC TCA CAA GCA TC	AAA AGC AGG ATT TCC AGA TCG
Interleukin 12A	IL12A	U14416	350	CAG CAA CAC GCT ACA GAA GG	CCT GCA TCA GCT CAG CAA TA
Interleukin 12B	IL12B	U11815	553	CCT GTC ACA AAG GAG GCG AGG C	CGT CAG GGA GAA GTA GGA ATG CGG
Interleukin 13	IL13	CB454692	477	CCT CAA TTT CTT CCT GTG CTG	ATG GTC AGG ATC TGC CTC TG
Interleukin 15	IL15	U42433	323	ATC CAG TGC TAC TTG TGT TTA C	GCT GTT TGC TAG CAT GGT AAG
MHC class II DYA	BOLA-DYA	AJ580583	623	ACC ACT TGG AAC AGC CAC TC	CAG TGC TCC ACT CTG CAG TC
Myeloid differentiation primary response gene	MYD88	AJ634627	431	GTG GTG GTG GTC TCT GAC G	GTG CCA CGT GTT CTA TGC AG
Nuclear factor of kappa light polypeptide gene enhancer in B-cells 2 (p49/p100)	NFKB2	CO259349	411	GGC TAT ACC CAG TCC ACC TG	TGA GGT CAA GAG GTG TGT GG
Prostaglandin-endoperoxide synthase 2	PTGS2	AF031698	651	CCA GAG CTC TTC CTC CTG TG	GCA GCT CTG GGT CAA ACT TC
Selectin P ligand (CD162)	SELPLG	BF654668	421	ACC ATC TTC CTC GTG TGC AC	CTA CCG GAG GTT CTG TTT GC
SH3-domain kinase binding protein 1	SH3KBP1	AJ819523	340	GTC AGG GCT TGA AAG GGA AG	GCC TTC TGT TCC TGC CAT AC
Signal transducer & activator or transcription 1	STAT1	BE588937	519	GCG AGT CAG ATC CAA GAT AG	ATC CTG AAG GTT ACG CTT GC
Tissue inhibitor of metalloproteinase 2	TIMP2	M32303	617	GGG CCA AAG CAG TCA ATA AG	TTC ATG CTG TGT CCA GGA AG
Toll-like receptor 7	TLR7	AY487802	693	GCC TTC TGA TTT CCA GCA TC	TTT GTC AGG CAC CTG ATT TG
Toll-like receptor 8	TLR8	AY642125	362	GGT GAT GAA TGA GCT GCG C	CTT TGC CAA AAC AAG CCC TC
Toll-like receptor 9	TLR9	AJ509825	694	TCA CCA GCC TCT CCT TAA TCT C	GAG AGA ACT GTC CTT CAA CAC C
Transforming growth factor β1	TGFB1	M36271	668	GCC ATA CTG GCC CTT TAC AA	GCT GAC GAA CAC AGC AGT TC
Transforming growth factor β3	TGFB3	CA034974	374	AGG TGT GGA AAT CAG CAT CC	CTG AGC AGA AGT TGG CAT AG
Transforming growth factor β receptor II	TGFBR2	CK772005	701	GTG TTG AGG GAG CCA TCT TC	AAC CTG CAG GAG TAC CTG AC
Tumour necrosis factor α	TNF	AF011926	660	AGC TGG CGG AGG AGG TGC TC	CAG GGC GAT GAT CCC AAA GTA GAC C
TNFRSF6 (fas)	FAS	U34794	645	TTC GGA AGA ACG GTA TGG AG	CCT GTG GAT AGG CAT GTG TG

List of the genes chosen for amplicon construction. The sequences used to design the primers and the sizes of the clone inserts are listed.

**Table 3:** Factors affecting intensity measurements.

Effect	Variance	SE (variance)	Variance %	Variance 0 hours	Variance 2 hours	Variance 16 hours	Variance 0 hours (%)	Variance 2 hours (%)	Variance 16 hours (%)
Slide	0.032	0.015	2	0.035	0.161	0.054	2	10	4
Gene	1.146	0.006	79	1.126	1.152	1.160	80	71	77
Replicate Spots	0.135	0.001	9	0.174	0.123	0.107	12	8	7
Channel	0.145	0.001	10	0.069	0.179	0.186	5	11	12

The contributions to variation in log(intensity) of slide, gene, replicate spots and channel, together with their standard errors and percentage variance accounted for. Equivalent estimates and percentages are presented separately for the 3 time points

**Table 4:** The top 25 genes exhibiting increased mRNA abundance 2 hours post activation.

Clone	E value	Accession No.	Gene Symbol	Gene	Fold Increase	FDRmax	P value
pIL8	0.0	NM_173925	IL8	Interleukin 8	40.00	1.23E-18	4.91E-22
MARC_3BOV_60P17	0.0	NM_174299	CXCL2	Chemokine (C-X-C motif) ligand 2 (GROβ/MIP2A)	38.20	1.25E-15	1.24E-18
C0006019e20	0.0	NM_181015	PRNP	Prion protein	28.13	1.05E-20	2.08E-24
MARC_1BOV_101M3	0.0	NM_174263	CCL20	Chemokine (C-C motif) ligand 20 (MIP3A)	25.81	3.14E-10	3.56E-12
MARC_1BOV_94L3	0.0	NM_174300	CXCL6	Chemokine (C-X-C motif) ligand 6	22.91	1.72E-11	1.23E-13
C0005209b8	6.00E-72	XM_612426	CCRN4L	CCR4 carbon catabolite repression 4-like	20.49	1.64E-11	1.14E-13
C0006018n14	0.0	NM_174006	CCL2	Chemokine (C-C motif) ligand 2 (MCP-1)	19.45	1.32E-07	4.19E-09
C0005204j16	0.0	XM_864683	CXCL3	Chemokine (C-X-C motif) ligand 3 (GROγ/MIP2b)	18.16	1.20E-13	3.40E-16
C0006020d11	0.0	XM_865242	ZFAND2A	Zinc finger, AN1-type domain 2A	17.61	1.10E-14	1.31E-17
C0006011e13	e-179	CK976904	-	EST	16.71	3.10E-08	8.41E-10
C0005208k22	e-167	NM_173198	NR4A3	Nuclear receptor subfamily 4, group A, member 3	15.65	2.39E-12	1.14E-14
C0006020e20	0.0	CB419326	-	EST	14.07	9.10E-12	5.60E-14
MARC_3BOV_36F6	0.0	NM_175827	CCL5	Chemokine (C-C motif) ligand 5 (RANTES)	13.84	2.90E-12	1.47E-14
C0006018g13	0.0	XM_611249	SLCO4A1	Solute carrier organic anion transporter family, member 4A1	13.79	4.62E-10	5.89E-12
MARC_1BOV_72M12	0.0	XM_866821	CXCL10	Chemokine (C-X-C motif) ligand 10 (IP10)	13.71	1.09E-05	6.09E-07
C0005199c13	0.0	AY248754	TNFAIP3	TNF-alpha induced protein 3	13.48	2.34E-08	5.90E-10
C0006015e11	0.0	XM_881561	PMAIP1	Phorbol-12-myristate-13-acetate-induced protein 1	12.99	1.95E-12	8.90E-15
C0005202i8	0.0	XM_583725	APOBEC3A	Apolipoprotein B mRNA editing enzyme, catalytic polypeptide-like 3A	12.96	1.20E-13	2.97E-16
MARC_3BOV_120N21	0.0	NM_174028	CSF3	Colony stimulating factor 3 (granulocyte) (G-CSF)	12.76	8.11E-11	7.73E-13
C0006015n15	2.00E-69	NM_002748	MAPK6	Mitogen-activated protein kinase 6 (erk-3)	12.64	8.88E-12	5.11E-14
C0006016h15	0.0	XM_613370	RGS1	Regulator of G-protein signalling 1	12.59	5.10E-09	1.00E-10
C0006013p15	e-158	CB534198	-	EST	12.59	1.93E-11	1.50E-13
C0005202d9	0.0	XM_870158	ANKRD22	Ankyrin repeat domain 22	11.99	6.36E-17	3.79E-20
MARC_1BOV_57B12	0.0	NM_174348	ICAM1	Intercellular adhesion molecule 1 (CD54)	11.40	6.40E-10	9.15E-12
C0005206b23	3.00E-82	NM_001432	EREG	Epiregulin	10.98	1.98E-07	6.61E-09

Summary of the top 25 genes exhibiting increased mRNA abundance 2 hours post activation, including clone name, accession number, e-value and identity of the best BLAST match and average fold change. The P and FDRmax values denote the probability from the moderated t-test and the Benjamini and Hochberg false discovery rate values respectively from the analysis of the resting cell values compared to the average activated sample values (Pre v Post).

**Table 5:** The top 25 genes exhibiting decreased mRNA abundance 2 hours post activation.

Clone	E value	Accession No.	Gene Symbol	Gene	Fold Decrease	FDRmax	P value
C0006018h16	0.0	NM_031483	ITCH	Itchy homolog E3 ubiquitin protein ligase	-5.36	2.55E-10	2.79E-12
MARC_2BOV_14N13	e-151	XM_582744	IL16	Interleukin 16	-4.94	9.35E-10	1.41E-11
C0006011p15	0.0	NM_003400	XPO1	Exportin 1	-4.11	3.28E-03	4.70E-04
C0006011d04	e-112	NM_000199	SGSH	N-sulfoglucosamine sulfohydrolase	-4.05	4.14E-05	2.88E-06
C0005202b11	0.0	NM_006565	CTCF	CCCTC-binding factor	-3.83	9.04E-06	4.90E-07
C0006014p20	0.0	NM_005642	TAF7	TAF7 RNA polymerase II, TATA box binding protein (TBP)-associated factor, 55kDa	-3.74	7.65E-09	1.61E-10
MARC_1BOV_107K4	0.0	XM_584158	CCR2	Chemokine (C-C motif) receptor 2	-3.70	4.81E-06	2.40E-07
C0005203a5	6.00E-54	NM_032804	C10orf22	Chromosome 10 open reading frame 22	-3.69	2.77E-04	2.68E-05
C0006005d04	0.0	XM_588702	UNG	Uracil-DNA glycosylase	-3.62	4.24E-10	5.00E-12
C0007385D18	0.0	BM087483	-	EST	-3.60	2.00E-03	2.63E-04
C0006007o03	e-153	NM_016046	EXOSC1	Exosome component 1	-3.51	6.69E-07	2.57E-08
C0006019m08	0.0	XM_868169	-	Hypothetical protein LOC616204	-3.51	8.67E-06	4.67E-07
C0005205c2	2.00E-70	NM_024939	RBM35B	RNA binding motif protein 35B	-3.50	7.22E-08	2.15E-09
C0005207d22	0.0	NM_013444	UBQLN2	Ubiquilin 2	-3.48	1.36E-08	3.13E-10
C0005198j2	7.00E-58	NM_139014	MAPK14	Mitogen-activated protein kinase 14 (p38)	-3.41	1.36E-08	3.15E-10
C0006005h08	0.0	NM_052911	ESCO1	Establishment of cohesion 1 homolog 1	-3.36	9.89E-06	5.48E-07
C0005202l16	4.00E-42	XM_545369	C6orf32	Chromosome 6 open reading frame 32	-3.32	2.43E-10	2.61E-12
MARC_2BOV_105E23	e-119	NM_153425	TRADD	TNFRSF1A-associated via death domain	-3.18	3.95E-07	1.45E-08
C0006007o20	e-111	NM_001001806	ZFP36L2	Zinc finger protein 36, C3H type-like 2	-3.18	3.22E-09	5.75E-11
C0005209h6	e-108	NM_006163	NFE2	Nuclear factor (erythroid-derived 2), 45kDa	-3.13	3.40E-06	3.27E-07
C0006019p19	e-106	XM_868699	TOB2	Transducer of ERBB2, 2	-3.13	8.37E-07	3.31E-08
C0005202g13	0.0	AV594592	-	EST	-3.11	7.31E-04	8.25E-05
C0005208j1	0.0	CB221804	-	EST	-3.10	6.65E-05	5.18E-06
C0006012f10	1.00E-67	NM_145037	FAM55C	Family with sequence similarity 55, member C	-3.08	1.03E-11	6.98E-14
C0005208h20	0.0	CB420023	-	EST	-3.01	1.67E-05	9.91E-07

Summary of the top 25 genes exhibiting decreased mRNA abundance 2 hours post activation, including clone name, accession number, e-value and identity of the best BLAST match and average fold change. The P and FDRmax values denote the probability from the moderated t-test and the Benjamini and Hochberg false discovery rate values respectively from the analysis of the resting cell values compared to the average activated sample values (Pre v Post).

**Table 6:** The top 25 genes exhibiting increased mRNA abundance 16 hours post activation.

Clone	E value	Accession No.	Gene Symbol	Gene	Fold Increase	FDRmax	P value
pIL8	0.0	NM_173925	IL8	Interleukin 8	60.09	1.23E-18	4.91E-22
MARC_1BOV_94L3	0.0	NM_174300	CXCL6	Chemokine (C-X-C motif) ligand 6	27.46	1.72E-11	1.23E-13
C0006007m23	2.00E-72	XM_868409	DFNA5	Deafness, autosomal dominant 5	25.42	6.52E-13	2.72E-15
MARC_3BOV_60P17	0.0	NM_174299	CXCL2	Chemokine (C-X-C motif) ligand 2 (GROβ/MIP2A)	25.14	1.25E-15	1.24E-18
C0006006k10	0.0	XM_586036	ANKRD37	Ankyrin repeat domain 37	23.35	4.05E-07	1.51E-08
C0006016h15	0.0	XM_613370	RGS1	Regulator of G-protein signalling 1	22.10	5.10E-09	1.00E-10
C0006019g12	e-134	CK965770	-	EST	21.24	2.26E-11	1.80E-13
C0006019e20	0.0	NM_181015	PRNP	Prion protein	20.34	1.05E-20	2.08E-24
C0006014j10	6.00E-96	NM_000214	JAG1	Jagged 1	18.39	1.81E-08	4.42E-10
C0005208m7	e-110	NM_000785	CYP27B1	Cytochrome P450, family 27, subfamily B, polypeptide 1	18.39	4.10E-09	7.83E-11
C0006007a22	0.0	NM_022154	SLC39A8	Solute carrier family 39 (zinc transporter), member 8	16.01	7.46E-10	1.10E-11
C0005204j16	0.0	XM_864683	CXCL3	Chemokine (C-X-C motif) ligand 3 (GROγ/MIP2b)	15.28	1.20E-13	3.40E-16
MARC_2BOV_43M14	e-132	NM_001124	ADM	Adrenomedullin	14.23	1.20E-13	3.00E-16
C0005198l1	e-138	BI975933	-	EST	11.26	2.12E-05	1.30E-06
MARC_3BOV_120N21	e-102	NM_172219	CSF3	Colony stimulating factor 3 (granulocyte) (G-CSF)	11.14	8.11E-11	7.73E-13
C0006020e20	0.0	CB419326	-	EST	10.79	9.10E-12	5.60E-14
MARC_1BOV_103H23	0.0	XM_586821	IL2RB	Interleukin 2 receptor, beta	9.96	3.73E-05	2.52E-06
C0006005p10	e-171	NM_001102	ACTN1	Actinin, alpha 1	9.95	7.22E-08	2.14E-09
C0006020m09	e-131	NM_005726	TSFM	Ts translation elongation factor, mitochondrial	9.63	5.24E-11	4.79E-13
C0006012n18	e-101	NM_000362	TIMP3	TIMP metallopeptidase inhibitor 3	9.50	2.71E-05	1.76E-06
C0005202d9	0.0	XM_870158	ANKRD22	Ankyrin repeat domain 22	9.48	6.36E-17	3.79E-20
pIL1B	0.0	NM_000576	IL1B	Interleukin 1, beta	9.37	3.16E-13	1.07E-15
C0006020d11	0.0	XM_865242	ZFAND2A	Zinc finger, AN1-type domain 2A	9.28	1.10E-14	1.31E-17
C0005208c5	e-100	NM_002575	SERPINB2	serpin peptidase inhibitor, clade B (ovalbumin), member 2	9.25	3.92E-14	6.22E-17
C0005202i8	0.0	XM_583725	APOBEC3A	Apolipoprotein B mRNA editing enzyme, catalytic polypeptide-like 3A	8.74	1.20E-13	2.97E-16

Summary of the top 25 genes exhibiting increased mRNA abundance 16 hours post activation including clone name, accession number, e-value and identity of the best BLAST match and average fold change. The P and FDRmax values denote the probability from the moderated t-test and the Benjamini and Hochberg false discovery rate values respectively from the analysis of the resting cell values compared to the average activated sample values (Pre v Post).

**Table 7:** The top 25 genes exhibiting decreased mRNA abundance 16 hours post activation.

Clone	E value	Accession No.	Gene Symbol	Gene	Fold Decrease	FDRmax	P value
C0006017i16	0.0	BM088889	-	EST	-22.29	2.39E-11	1.94E-13
C0005199l3	0.0	BT021551	RNASE4	Ribonuclease, RNase A family, 4	-21.08	1.43E-09	2.33E-11
C0005209g19	-	-	-	Unique singleton	-13.17	5.49E-13	2.07E-15
C0005198n19	0.0	NM_000014	A2M	Apha-2-macroglobulin	-12.22	6.56E-07	2.50E-08
C0006007m24	1.00E-48	NM_174594	RNASE6	Ribonuclease k6	-8.88	9.11E-12	5.79E-14
C0006020f22	0.0	XM_868762	CLEC4A	C-type lectin domain family 4, member A	-8.45	2.80E-06	1.27E-07
C0005202l16	4.00E-42	XM_545369	C6orf32	Chromosome 6 open reading frame 32	-8.21	2.43E-10	2.61E-12
BARC_5BOV_46G22	0.0	NM_001037446	CD59	CD59 antigen	-8.15	2.11E-07	7.20E-09
C0005206d13	4.00E-57	NM_001009567	MRC1L1	Mannose receptor, C type 1-like 1	-8.15	5.55E-10	7.83E-12
C0005198i18	1.00E-34	AY112657	FGL2	Fibrinogen-like 2	-8.04	5.69E-07	2.16E-08
C0006012b04	0.0	DY139256	-	EST	-6.79	8.65E-10	1.29E-11
C0005206a23	e-163	CK954547	-	EST	-6.34	1.27E-06	5.27E-08
C0005920C16	0.0	DN742393	-	EST	-6.12	7.62E-08	2.29E-09
MARC_3BOV_43C11	e-101	NM_172087	TNFSF13	Tumor necrosis factor (ligand) superfamily, member 13 (APRIL)	-5.81	1.51E-08	3.57E-10
C0005201e6	e-100	NM_005923	MAP3K5	Mitogen-activated protein kinase kinase kinase 5	-5.65	5.83E-04	6.40E-05
C0006018f03	2.00E-41	DV230110	-	EST	-5.55	3.13E-06	1.46E-07
C0006005m10	e-121	NM_001031733	CALML4	Calmodulin-like 4	-5.47	2.06E-07	6.98E-09
C0005210k15	0.0	XM_613642	IQGAP2	IQ motif containing GTPase activating protein 2	-5.45	1.58E-07	5.13E-09
C0005199n6	0.0	NM_001033990	SCP2	Sterol carrier protein 2	-5.44	3.32E-09	5.99E-11
C0005209n7	7.00E-95	NM_001386	DPYSL2	Dihydropyrimidinase-like 2	-5.16	8.42E-08	2.58E-09
MARC_2BOV_14N13	e-151	XM_582744	IL16	Interleukin 16	-5.12	9.35E-10	1.41E-11
C0006016k03	e-124	XM_607564	FLI1	Friend leukemia virus integration 1	-4.88	1.90E-05	1.14E-06
C0005209m4	0.0	XM_583952	IL6R	Interleukin 6 receptor	-4.88	2.49E-11	2.08E-13
MARC_2BOV_42H5	0.0	NM_001014945	C1QA	Complement component 1, q subcomponent, alpha	-4.86	8.14E-06	4.32E-07
C0006018i05	0.0	XM_615814	FKBP5	FK506 binding protein 5	-4.65	7.14E-09	1.46E-10

Summary of the top 25 genes exhibiting decreased mRNA abundance 16 hours post activation, including clone name, accession number, e-value and identity of the best BLAST match and average fold change. The P and FDRmax values denote the probability from the moderated t-test and the Benjamini and Hochberg false discovery rate values respectively from the analysis of the resting cell values compared to the average activated sample values (Pre v Post).

**Table 8:** Details of the qRT-PCR primers and amplicons.

Gene	Gene Symbol	Clone	Direction	Sequence (5'-3')	Size (bp)
CD9 antigen	CD9	BE751736	F	TTG GAC TAT GGC TCC GAT TC	261
			R	CCT TGA TCA CCT CCT CCT TG	
CD44 antigen	CD44	AJ816969	F	TAC CCA CCC ACA AGA GAA CC	102
			R	AAC CCA TTA CCA GTC ATC CTT G	
CD86 antigen	CD86	pCD86	F	GAA GGT CCC AAG GAT TGG TT	136
			R	GAT GAG CAG GTC AAA TTG ATG A	
Chemokine (C-C motif) ligand 2	CCL2	AJ821161	F	TCG CCT GCT GCT ATA CAT TC	274
			R	AGA GGG CAG TTA GGG AAA GC	
Chemokine (C-C motif) receptor 1	CCR1	BE756795	F	AAA TGA GAA GAA GGC CAA AGC	140
			R	TGC TCT GCT CAC ACT TAC GG	
Complement component 1, r subcomponent	C1R	AJ818500	F	AGA TTT GTC CGT CTG CCA GT	183
			R	CCC AGC GGT CAT TTC TCT TA	
Fibronectin 1	FN1	AJ819936	F	TAT CTT GGT GCC ATT TGC TC	258
			R	ATC CAC GCT TGT TTC TCT GG	
Interleukin 6	IL6	pIL6	F	TGA GTG TGA AAG CAG CAA GG	289
			R	CAG CAG GTC AGT GTT TGT GG	
Prion protein	PRNP	AJ821011	F	TGT GTA TGG TGG GAT TGG AA	197
			R	GCT GGG TCA TCA AAG AAA TG	
V-fos FBJ murine osteosarcoma viral oncogene	FOS	BE755117	F	CTT CTC CAG CAT GGG TTC TC	257
			R	TCT GAG CTC TGC CTC CTG TC	
Chromosome 13 open reading frame 8	C13orf8	AJ817183	F	AGC AGT GAC CAA GAG CAG GT	205
			R	TCA TAG CAC GAC AGC AAC AA	

F and R denote forward and reverse primers respectively.

## References

[B1] Glass EJ, Innes EA, Spooner RL, Brown CG (1989). Infection of bovine monocyte/macrophage populations with *Theileria annulata *and *Theileria parva*. Vet Immunol Immunopathol.

[B2] Bogdan C, Röllinghoff M (1999). How do protozoan parasites survive inside macrophages?. Parasitol Today.

[B3] McGuire K, Glass EJ (2005). The expanding role of microarrays in the investigation of macrophage responses to pathogens. Vet Immunol Immunopathol.

[B4] Chitko-McKown CG, Fox JM, Miller LC, Heaton MP, Bono JL, Keen JE, Grosse WM, Laegreid WW (2004). Gene expression profiling of bovine macrophages in response to *Escherichia coli *0157:H7 lipopolysaccharide. Dev Comp Immunol.

[B5] Weiss DJ, Evanson OA, Deng M, Abrahamsen MS (2004). Gene expression and antimicrobial activity of bovine macrophages in response to *Mycobacterium avium *subsp. *paratuberculosis*. Vet Pathol.

[B6] Weiss DJ, Evanson OA, Deng MQ, Abrahamsen MS (2004). Sequential patterns of gene expression by bovine monocyte-derived macrophages associated with ingestion of mycobacterial organisms. Microb Pathogenesis.

[B7] Werling D, Ruryk A, Heaney J, Moeller E, Brownlie J (2005). Ability to differentiate between cp and ncp BVDV by microarrays: Towards an application in clinical veterinary medicine?. Vet Immunol Immunopathol.

[B8] Wedlock DN, Kawakami RP, Koach J, Buddle BM, Collins DM (2006). Differences of gene expression in bovine alveolar macrophages infected with virulent and attenuated isogenic strains of *Mycobacterium bovis*. Int Immunopharmacol.

[B9] Staudt LM, Brown PO (2000). Genomic views of the immune system. Annu Rev Immunol.

[B10] Wells C, Ravasi T, Sultana R, Yagi K, Carninci P, Bono H, Faulkner G, Okazaki Y, Quackenbush J, Hume DA, Lyons PA (2003). Continued discovery of transcriptional units expressed in cells of the mouse mononuclear phagocyte lineage. Genome Res.

[B11] Kawai J, Shinagawa A, Shibata K, Yoshino M, Itoh M, Ishii Y, Arakawa T, Hara A, Fukunishi Y, Konno H, Adachi J, Fukuda S, Aizawa K, Izawa M, Nishi K, Kiyosawa H, Kondo S, Yamanaka I, Saito T, Okazaki Y, Gojobori T, Bono H, Kasukawa T, Saito R, Kadota K, Matsuda H, Ashburner M, Batalov S, Casavant T, Fleischmann W, Gaasterland T, Gissi C, King B, Kochiwa H, Kuehl P, Lewis S, Matsuo Y, Nikaido I, Pesole G, Quackenbush J, Schriml LM, Staubli F, Suzuki R, Tomita M, Wagner L, Washio T, Sakai K, Okido T, Furuno M, Aono H, Baldarelli R, Barsh G, Blake J, Boffelli D, Bojunga N, Carninci P, de Bonaldo MF, Brownstein MJ, Bult C, Fletcher C, Fujita M, Gariboldi M, Gustincich S, Hill D, Hofmann M, Hume DA, Kamiya M, Lee NH, Lyons P, Marchionni L, Mashima J, Mazzarelli J, Mombaerts P, Nordone P, Ring B, Ringwald M, Rodriguez I, Sakamoto N, Sasaki H, Sato K, Schonbach C, Seya T, Shibata Y, Storch KF, Suzuki H, Toyo-oka K, Wang KH, Weitz C, Whittaker C, Wilming L, Wynshaw-Boris A, Yoshida K, Hasegawa Y, Kawaji H, Kohtsuki S, Hayashizaki Y (2001). Functional annotation of a full-length mouse cDNA collection. Nature.

[B12] Okazaki Y, Furuno M, Kasukawa T, Adachi J, Bono H, Kondo S, Nikaido I, Osato N, Saito R, Suzuki H, Yamanaka I, Kiyosawa H, Yagi K, Tomaru Y, Hasegawa Y, Nogami A, Schonbach C, Gojobori T, Baldarelli R, Hill DP, Bult C, Hume DA, Quackenbush J, Schriml LM, Kanapin A, Matsuda H, Batalov S, Beisel KW, Blake JA, Bradt D, Brusic V, Chothia C, Corbani LE, Cousins S, Dalla E, Dragani TA, Fletcher CF, Forrest A, Frazer KS, Gaasterland T, Gariboldi M, Gissi C, Godzik A, Gough J, Grimmond S, Gustincich S, Hirokawa N, Jackson IJ, Jarvis ED, Kanai A, Kawaji H, Kawasawa Y, Kedzierski RM, King BL, Konagaya A, Kurochkin IV, Lee Y, Lenhard B, Lyons PA, Maglott DR, Maltais L, Marchionni L, McKenzie L, Miki H, Nagashima T, Numata K, Okido T, Pavan WJ, Pertea G, Pesole G, Petrovsky N, Pillai R, Pontius JU, Qi D, Ramachandran S, Ravasi T, Reed JC, Reed DJ, Reid J, Ring BZ, Ringwald M, Sandelin A, Schneider C, Semple CA, Setou M, Shimada K, Sultana R, Takenaka Y, Taylor MS, Teasdale RD, Tomita M, Verardo R, Wagner L, Wahlestedt C, Wang Y, Watanabe Y, Wells C, Wilming LG, Wynshaw-Boris A, Yanagisawa M, Yang I, Yang L, Yuan Z, Zavolan M, Zhu Y, Zimmer A, Carninci P, Hayatsu N, Hirozane-Kishikawa T, Konno H, Nakamura M, Sakazume N, Sato K, Shiraki T, Waki K, Kawai J, Aizawa K, Arakawa T, Fukuda S, Hara A, Hashizume W, Imotani K, Ishii Y, Itoh M, Kagawa I, Miyazaki A, Sakai K, Sasaki D, Shibata K, Shinagawa A, Yasunishi A, Yoshino M, Waterston R, Lander ES, Rogers J, Birney E, Hayashizaki Y (2002). Analysis of the mouse transcriptome based on functional annotation of 60,770 full-length cDNAs. Nature.

[B13] Jensen K, Speed D, Paxton E, Williams JL, Glass EJ (2006). Construction of a normalized *Bos taurus *and *Bos indicus *macrophage specific cDNA library. Anim Genet.

[B14] Iseli C, Jongeneel CV, Bucher P (1999). ESTScan: a program for detecting, evaluating, and reconstructing potential coding regions in EST sequences. P Int Conf Intell Syst Mol Biol.

[B15] ESTScan. http://www.ch.embnet.org/software/ESTScan.html.

[B16] Smith TP, Grosse WM, Freking BA, Roberts AJ, Stone RT, Casas E, Wray JE, White J, Cho J, Fahrenkrug SC, Bennett GL, Heaton MP, Laegreid WW, Rohrer GA, Chitko-McKown CG, Pertea G, Holt I, Karamycheva S, Liang F, Quackenbush J, Keele JW (2001). Sequence evaluation of four pooled-tissue normalized bovine cDNA libraries and construction of a gene index for cattle. Genome Res.

[B17] Sonstegard TS, Capuco AV, White J, Van Tassell CP, Connor EE, Cho J, Sultana R, Shade L, Wray JE, Wells KD, Quackenbush J (2002). Analysis of bovine mammary gland EST and functional annotation of the *Bos taurus *gene index. Mamm Genome.

[B18] ARK-Genomics Centre for Functional Genomics in Farm Animals. http://www.ark-genomics.org.

[B19] Brazma A, Parkinson H, Sarkans U, Shojatalab M, Vilo J, Abeygunawardena N, Holloway E, Kapushesky M, Kemmeren P, Lara GG, Oezcimen A, Rocca-Serra P, Sansone S (2003). ArrayExpress – a public repository for microarray gene expression data at the EBI. Nucleic Acids Res.

[B20] ArrayExpress. http://www.ebi.ac.uk/arrayexpress.

[B21] Tempelman RJ (2005). Assessing statistical precision, power and robustness of alternative experimental designs for two colour microarray platforms based on mixed effects models. Vet Immunol Immunopathol.

[B22] Yao J, Burton JL, Saama P, Sipkovsky S, Coussens PM (2001). Generation of EST and cDNA microarray resources for the study of bovine immunobiology. Acta Vet Scand.

[B23] Hill EW, O'Gorman GM, Agaba M, Gibson JP, Hanotte O, Kemp SJ, Naessens J, Coussens PM, MacHugh DE (2005). Understanding bovine trypanosomiasis and trypanotolerance: the promise of functional genomics. Vet Immunol Immunopathol.

[B24] Tao W, Mallard BA, Karrow N, Bridle B (2004). Construction and application of a bovine immune-endocrine cDNA microarray. Vet Immunol Immunopathol.

[B25] Donaldson L, Vuocolo T, Gray C, Strandberg Y, Reverter A, McWilliam S, Wang YH, Byrne K, Tellam R (2005). Construction and validation of a bovine innate immune microarray. BMC Genomics.

[B26] Band MR, Olmstead C, Everts RE, Liu ZL, Lewin HA (2002). A 3800 gene microarray for cattle functional genomics: comparison of gene expression in spleen, placenta, and brain. Anim Biotechnol.

[B27] Gao JJ, Diesl V, Wittmann T, Morrison DC, Ryan JL, Vogel SN, Follettie MT (2003). Bacterial LPS and CpG DNA differentially induce gene expression profiles in mouse macrophages. J Endotoxin Res.

[B28] Nau GJ, Richmond JFL, Schlesinger A, Jennings EG, Lander ES, Young RA (2002). Human macrophage activation programs induced by bacterial pathogens. P Natl Acad Sci USA.

[B29] Chaussabel D, Semnani RT, McDowell MA, Sacks D, Sher A, Nutman TB (2003). Unique gene expression profiles of human macrophages and dendritic cells to phylogenetically distinct parasites. Blood.

[B30] Wang Y, Barbacioru C, Hyland F, Xiao W, Hunkapiller KL, Blake J, Chan F, Gonzalez C, Zhang L, Samaha RR (2006). Large scale real-time PCR validation on gene expression measurements from two commercial long-oligonucleotide microarrays. BMC Genomics.

[B31] Evertsz EM, Au-Young J, Ruvolo MV, Lim AC, Reynolds MA (2001). Hybridization cross-reactivity within homologous gene families on glass cDNA microarrays. Biotechniques.

[B32] Patel OV, Suchyta SP, Sipkovsky SS, Yao J, Ireland JJ, Coussens PM, Smith GW (2005). Validation and application of a high fidelity mRNA linear amplification procedure for profiling gene expression. Vet Immunol Immunopathol.

[B33] Rouzaut A, Lopez-Moratalla N, de Miguel C (2000). Differential gene expression in the activation and maturation of human monocytes. Arch Biochem Biophys.

[B34] Zhou AQ, Herriott MJ, Leu RW (1991). Kinetics of the biosynthesis of complement subcomponent C1q by murine macrophages: LPS, immune complexes, and zymosan alone and in combination with interferon-gamma. J Leukocyte Biol.

[B35] Dürig J, Giese A, Schulz-Schaeffer W, Rosenthal C, Schmücker U, Bieschke J, Dührsen U, Kretzschmar HA (2000). Differential constitutive and activation-dependent expression of prion protein in human peripheral blood leucocytes. Br J Haematol.

[B36] de Almeida CJG, Chiarini LB, da Silva JP, e Silva PMR, Martins MA, Linden R (2005). The cellular prion protein modulates phagocytosis and inflammatory response. J Leukocyte Biol.

[B37] Burthem J, Urban B, Pain A, Roberts DJ (2001). The normal cellular prion protein is strongly expressed by myeloid dendritic cells. Blood.

[B38] Sun Y, Leaman DW (2005). Involvement of noxa in cellular apoptotic responses to interferon, double-stranded RNA, and virus infection. J Biol Chem.

[B39] Bogerd HP, Wiegard HL, Doehle BP, Lueders KK, Cullen BR (2006). APOBEC3A and APOBEC3B are potent inhibitors of LTR-retrotransposan function in human cells. Nucleic Acids Res.

[B40] Scheibenbogen C, Keiholz U, Richter M, Andreesen R, Hunstein W (1992). The interleukin-2 receptor in human monocytes and macrophages: regulation of expression and release of the alpha and beta chains (p55 and p75). Res Immunol.

[B41] Dekkers PEP, Juffermans NP, ten Hove T, de Jonge E, van Deventer SJH, van der Poll T (2000). Endotoxin down-regulates monocyte and granulocyte interleukin-6 receptors without influencing gp130 expression in humans. J Infect Dis.

[B42] Parker LC, Whyte MKB, Vogel SN, Dower SK, Sabroe I (2004). Toll-like receptor (TLR)2 and TLR4 agonists regulate CCR expression in human monocytic cells. J Immunol.

[B43] Grey ST, Tsuchida A, Hau H, Orthner CL, Salem HH, Hancock WW (1994). Selective inhibitory effects of the anticoagulant activated protein C on the responses of human mononuclear phagocytes to LPS, IFN-γ, or phorbol ester. J Immunol.

[B44] Pei L, Castrillo A, Chen M, Hoffmann A, Tontonoz P (2005). Induction of NR4A orphan nuclear receptor expression in macrophages in response to inflammatory stimuli. J Biol Chem.

[B45] Begum NA, Kobayashi M, Moriwaki Y, Matsumoto M, Toyoshima K, Seya T (2002). *Mycobacterium bovis *BCG cell wall and lipopolysaccharide induce a novel gene, BIGM103, encoding a 7-TM protein: identification of a new protein family having Zn-transporter and Zn-metalloprotease signatures. Genomics.

[B46] Rozen S, Skaletsky HJ, Krawetz S, Misener S (2000). Primer3 on the WWW for general users and for biologist programmers. Bioinformatics Methods and Protocols: Methods in Molecular Biology.

[B47] Primer3. http://frodo.wi.mit.edu/cgi-bin/primer3/primer3_www.cgi.

[B48] Brooke GP, Parsons KR, Howard CJ (1998). Cloning of two members of the SIRPα family of protein tyrosine phosphatase binding proteins in cattle that are expressed on monocytes and a sub-population of dendritic cells and which mediate binding to CD4 T cells. Eur J Immunol.

[B49] Smyth GK, Gentleman R, Carey V, Dudoit S, Irizarry R, Huber (2005). Limma: linear models for microarray data. Bioinformatics and Computational Biology Solutions using R and Bioconductor.

[B50] Smyth GK, Speed TP (2003). Normalization of cDNA microarray data. Methods.

[B51] Baird D, Johnstone P, Wilson T (2004). Normalization of microarray data using a spatial mixed model analysis which includes splines. Bioinformatics.

[B52] Smyth GK (2004). Linear models and empirical Bayes methods for assessing differential expression in microarray experiments. Statistical Applications in Genetics and Molecular Biology.

[B53] Benjamini Y, Hochberg Y (1995). Controlling the false discovery rate: a practical and powerful approach to multiple testing. J Roy Stat Soc B Met.

[B54] NetPrimer. http://www.premierbiosoft.com/netprimer/netprlaunch/netprlaunch.html.

[B55] Pfaffl MW (2001). A new mathematical model for relative quantification in real-time RT-PCR. Nucleic Acids Res.

[B56] Roslin Institute. http://www.roslin.ac.uk/about/staff/Bovine_Macrophage_cDNA_Microarray.xls.

